# A genome-wide investigation into the underlying genetic architecture of personality traits and overlap with psychopathology

**DOI:** 10.1038/s41562-024-01951-3

**Published:** 2024-08-12

**Authors:** Priya Gupta, Marco Galimberti, Yue Liu, Sarah Beck, Aliza Wingo, Thomas Wingo, Keyrun Adhikari, Henry R. Kranzler, VA Million Veteran Program, Priya Gupta, Marco Galimberti, Sarah Beck, Henry R. Kranzler, Murray B. Stein, Joel Gelernter, Daniel F. Levey, Murray B. Stein, Joel Gelernter, Daniel F. Levey

**Affiliations:** 1 Division of Human Genetics, Department of Psychiatry, Yale University School of Medicine, New Haven, CT USA; 2 Department of Psychiatry, Veterans Affairs Connecticut Healthcare Center, West Haven, CT USA; 3 Department of Neurology and Human Genetics, Emory University School of Medicine, Atlanta, GA USA; 4 Department of Psychiatry and Behavioral Sciences, Emory University School of Medicine, Atlanta, GA USA; 5 Atlanta Veterans Affairs Medical Center, Atlanta, GA USA; 6 Crescenz Veterans Affairs Medical Center, Philadelphia, PA USA; 7 Department of Psychiatry, University of Pennsylvania Perelman School of Medicine, Philadelphia, PA USA; 8 Psychiatry Service, VA San Diego Healthcare System, San Diego, CA USA; 9 Departments of Psychiatry, School of Medicine, and Herbert Wertheim School of Public Health, University of California San Diego, La Jolla, CA USA

**Keywords:** Genetic variation, Human behaviour

## Abstract

Personality is influenced by both genetic and environmental factors and is associated with other psychiatric traits such as anxiety and depression. The ‘big five’ personality traits, which include neuroticism, extraversion, agreeableness, conscientiousness and openness, are a widely accepted and influential framework for understanding and describing human personality. Of the big five personality traits, neuroticism has most often been the focus of genetic studies and is linked to various mental illnesses, including depression, anxiety and schizophrenia. Our knowledge of the genetic architecture of the other four personality traits is more limited. Here, utilizing the Million Veteran Program cohort, we conducted a genome-wide association study in individuals of European and African ancestry. Adding other published data, we performed genome-wide association study meta-analysis for each of the five personality traits with sample sizes ranging from 237,390 to 682,688. We identified 208, 14, 3, 2 and 7 independent genome-wide significant loci associated with neuroticism, extraversion, agreeableness, conscientiousness and openness, respectively. These findings represent 62 novel loci for neuroticism, as well as the first genome-wide significant loci discovered for agreeableness. Gene-based association testing revealed 254 genes showing significant association with at least one of the five personality traits. Transcriptome-wide and proteome-wide analysis identified altered expression of genes and proteins such as *CRHR1, SLC12A5, MAPT* and *STX4*. Pathway enrichment and drug perturbation analyses identified complex biology underlying human personality traits. We also studied the inter-relationship of personality traits with 1,437 other traits in a phenome-wide genetic correlation analysis, identifying new associations. Mendelian randomization showed positive bidirectional effects between neuroticism and depression and anxiety, while a negative bidirectional effect was observed for agreeableness and these psychiatric traits. This study improves our comprehensive understanding of the genetic architecture underlying personality traits and their relationship to other complex human traits.

## Abstract (web summary)

Using genome-wide association studies and meta-analyses on dimensions of personality from large existing datasets, Gupta et al. find novel genomic loci that refine our understanding of the genetic architecture of complex traits.

## Main

Personality dimensions influence behaviour, thoughts, feelings and reactions to different situations. A valuable construct within the field of psychological research has converged on five different dimensions to characterize human personality: neuroticism, extraversion, agreeableness, conscientiousness and openness^1,2^. Personality dimensions could be playing an important role in the susceptibility and resilience to diagnosis of psychiatric disorders and their relationship with other health-related traits and responses to treatment.

The last decade has seen an increasing interest in understanding the dimensions of human personality through the lens of genetics. Depression is one mental disorder that has been studied with respect to its relationship to personality traits, with a large portion of genetic risk for depression being captured by neuroticism^3^. The same study found a modest negative association of genetic depression risk with conscientiousness, with small contributions from openness, agreeableness and extraversion. Neuroticism is one of the most studied dimensions of the ‘big five’ personality traits and numerous studies have found positive correlations with depression, anxiety and other mental illnesses^3–5^. Schizophrenia has also been associated with personality traits, especially neuroticism, which has been shown to increase risk for diagnosis^6^. A study using data from the Psychiatric Genomics Consortium (PGC) and personal genomics company 23andMe found two genomic loci to be common between neuroticism and schizophrenia. This study also reported six loci shared between schizophrenia and openness^7^.

The past 15 years have seen an explosion in the use of the genome-wide association study (GWAS). In 2010, Marleen de moor et al. from the Genetics of Personality Consortium (GPC) published a GWAS of the ‘big five’ personality traits conducted with 17,375 adults from 15 different samples of European ancestry (EUR)^8^. This study found two genome-wide significant (GWS) variants near the *RASA1* gene on 5q14.3 for openness and one near *KATNAL2* on 18q21.1 for conscientiousness but no significant associations for other personality traits. GPC then conducted studies on extraversion and neuroticism in their second phase and meta-analyses were performed. A GWAS of neuroticism that was conducted on approximately 73,000 subjects identified rs35855737 in the *MAG1* gene as a GWS variant^9^. Although the sample size was increased substantially to 63,030 subjects in phase II, no GWS variants were detected for extraversion in that study^10^. In 2016, Lo et al. identified six loci associated with different personality traits, including loci for extraversion^11^. A paper that investigated neuroticism along with subjective well-being and depressive symptoms leveraging the UK Biobank (UKB) and other published data^12^ was published this same year. A more detailed picture of neuroticism genetics was presented by Nagel et al. 2018^13^, where the authors collected neuroticism genotype data of 372,903 individuals from the UKB and performed a meta-analysis by combining the summary statistics from this UKB sample, 23andMe and GPC phase 1 samples, increasing the total sample size to 449,484. They identified a total of 136 loci and 599 genes showing GWS associations to neuroticism. In 2021, Becker et al. conducted a polygenic index study and created a resource with GWAS meta-analysis summary statistics combining different data cohorts for a large number of traits, including neuroticism, thus increasing the total sample size of neuroticism meta-analysis to 484,560 and increasing the number of novel GWS loci (although this was not the focus of this work)^14^. They also identified six genomic loci for extraversion.

In this work, we conducted GWAS of each of the ‘big five’ personality traits in a sample of ~224,000 individuals with genotype data available from the Million Veteran Program (MVP). Using linkage disequilibrium score regression (LDSC), we estimated the single-nucleotide polymorphism (SNP)-based heritability of each of the five personality traits. We then combined the MVP data with other sources of personality GWAS summary statistics from GPC and UKB and performed meta-analyses for each of the five personality traits, including as many as ~680,000 participants for the largest meta-analysis of neuroticism so far. To gain insights into the biology of these traits, we performed transcriptome-wide association studies (TWAS) and proteome-wide association studies (PWAS) followed by pathway and drug perturbation analyses and variant fine-mapping. We also studied the overlap of these personality traits with anxiety and other complex traits through phenome-wide genetic correlations and conditional analyses. We performed drug perturbation analyses with genes associated with neuroticism and found convergence on drugs for major depressive disorder (MDD). Finally, we conducted Mendelian randomization (MR) experiments to investigate the causal relationship of neuroticism and agreeableness, the two most genetically divergent traits, with depression and anxiety.

## Results

### MVP GWAS

In the EUR GWAS in the MVP cohort, we identified in total 34 unique independent genomic loci significantly associated (*P* value <5 × 10^−8^) with at least one of the five personality traits (Table 1). The highest numbers of loci were found for extraversion and neuroticism (11 for each) while conscientiousness showed only two loci. In the MVP we identified 4,036 GWS variants (*P* < 5 × 10^−8^) for neuroticism across 7 independent genomic loci harbouring genes including *MAD1L1*, *MAP3K14*, *CRHR1*, *CRHR1-IT1* and *VK2* (*P* < 5 × 10^−8^). Of these seven loci, two (rs2717043 and rs4757136) were also reported to be GWS in Nagel et al.^13^. We identified 11 GWS loci for extraversion, the largest number of GWS loci to be identified for this trait. Associations for extraversion were found near several genes, including *CRHR1*, *MAPT* and *METTL15* (total 90 genes). For the two conscientiousness loci, the first locus maps to a region near the genes *FOXP2*, *PPP1R3A* and *MDFIC* and the second locus maps to the *ZNF704* gene, all of which are protein coding genes. For openness, 7 loci were identified spanning over 39 genes, including *BRMS1*, *RIN1* and B3GNT1. For agreeableness, 3 loci were identified spanning 19 genes, including *SOX7*, *PINX1* and *FOXP2*. The Manhattan plots for all five traits are shown in Supplementary Fig. 1.

**Table 1:** Genomic loci identified in the MVP cohort for the five personality traits

Lead SNP	Position	Effect size	s.e.m.	*P*	Gene
**Neuroticism**					
rs4129585	8:143312933:C:A	−0.05241	0.006589	1.80027 × 10^−15^	TSNARE1
rs574307253	17:43667635:G:A	0.059817	0.007909	3.95325 × 10^−14^	–
rs116956554	17:44699851:A:G	0.057427	0.008282	4.09437 × 10^−12^	NSF
rs2001433	8:10903475:A:T	0.046409	0.006702	4.38959 × 10^−12^	XKR6
rs7396943	11:13328979:C:G	0.046283	0.006822	1.16916 × 10^−11^	BMAL1
rs7825636	8:8578229:G:C	0.04446	0.006712	3.49922 × 10^−11^	–
rs6948912	7:2076701:T:C	−0.04524	0.007191	3.14987 × 10^−10^	MAD1L1
rs2139053	2:58156539:C:T	0.042452	0.006905	7.86894 × 10^−10^	–
rs615632	8:9796321:T:C	0.040914	0.00673	1.20538 × 10^−9^	–
rs117713019	8:143444050:T:C	0.08952	0.015421	6.4366 × 10^−9^	TSNARE1
rs6498809	16:61833811:T:C	0.03672	0.006642	3.23728 × 10^−8^	CDH8
**Extraversion**					
rs35424804	11:13248730:T:C	0.037166	0.005994	5.6352 × 10^−10^	–
rs17688916	17:43778680:A:T	−0.04406	0.007384	2.41754 × 10^−9^	–
rs12971383	19:31876692:C:A	0.054198	0.009231	4.33065 × 10^−9^	–
rs3764002	12:108618630:T:C	−0.03928	0.006717	4.97048 × 10^−9^	WSCD2
rs1011501	5:93008722:C:T	−0.03858	0.006619	5.60839 × 10^−9^	FAM172A
rs35918640	17:79452756:AT:A	0.034989	0.006064	7.93604 × 10^−9^	–
rs5831479	2:58167698:G:GA	−0.03516	0.006154	1.10698 × 10^−8^	–
rs11209774	1:71834574:G:T	−0.03406	0.005964	1.12553 × 10^−8^	–
rs7606514	2:185130889:G:A	−0.04297	0.007632	1.79308 × 10^−8^	–
rs7739331	6:92315317:T:G	−0.03211	0.005872	4.5495 × 10^−8^	−
rs1444978	4:85363489:C:T	0.036666	0.0067175	4.81211 × 10^−8^	−
**Agreeableness**					
rs17137124	7:114210814:C:T	−0.03273	0.005234	3.99629 × 10^−10^	FOXP2
rs7833945	8:10700266:G:T	−0.03161	0.005375	4.05674 × 10^−9^	−
rs7240986	18:53195249:A:G	−0.0307	0.005464	1.92256 × 10^−8^	TCF4
**Conscientiousness**					
rs78446248	8:81443461:A:G	−0.08581	0.014907	8.61644 × 10^−9^	−
rs936145	7:114297180:A:G	−0.0331	0.005848	1.50791 × 10^−8^	FOXP2
**Openness**					
rs7570	11:66610645:C:G	−0.04711	0.006439	2.54365 × 10^−13^	C11orf80
rs117890891	6:135928772:G:T	0.145052	0.023233	4.28749 × 10^−10^	−
rs919013	4:152945667:C:T	0.033444	0.005625	2.7658 × 10^−9^	−
rs6996198	8:65463442:T:C	−0.04602	0.007788	3.43929 × 10^−9^	−
rs6725323	2:29377923:T:A	−0.03361	0.005991	2.03021 × 10^−8^	CLIP4
rs61689447	9:35777442:G:G:TT	−0.03312	0.005946	2.55878 × 10^−8^	−
rs11996715	8:141647291:A:C	0.030703	0.005588	3.91526 × 10^−8^	−

Two GWS variants were found for agreeableness in the African ancestry (AFR) sample. Variants rs2393573 (effect size, −0.106; standard error of the mean (s.e.m.), 0.018; 95% confidence interval (CI) −0.071, 0.141; *P* = 7.502 × 10^−9^) and rs112726823 (effect, −0.720; s.e.m., 0.130; 95% CI 0.465, 0.975; *P* 3.268 × 10^−8^) mapped near *CCDC6* and *ARHGAP24*. We did not find any GWS variants for any of the other four personality traits in the AFR sample; the multiple subthreshold findings from this analysis may reach the GWS threshold in a larger sample. A list of lead independent SNPs found in the AFR sample for each trait is provided in Supplementary Tables 1–5.

### Meta-analysis in EUR populations

The meta-analysis for neuroticism showed associations with 208 independent GWS loci. The increased power due to the inclusion of MVP data resulted in the identification of 79 additional GWS loci, which were not significant in the previous study^13^. Only five loci identified previously (rs1763839, rs2295094, rs11184985, rs579017 and rs76923064) were no longer significant in our meta-analysis. A total of 17 loci of these 79 have also been discovered in the polygenic index study (Supplementary Table 6). Thus, we found 62 novel loci associated with neuroticism in our meta-analysis. SNPs and loci were mapped to genes based on chromosomal position, expression quantitative trait loci (eQTL) and chromatic interaction^15^. A total of 231 genes were found significant in the MAGMA (Multi-marker Analysis of GenoMic Annotation) gene-based test^16^. *NSF*, *KANSL1*, *FMNL1*, *PLEKHM1* and *CRHR1* (*P* < 2.850 × 10^−40^) were among the top significant hits. The largest number of significant loci are located on chromosome 11, followed by chromosome 1. The GWS associations also include two loci with variants rs7818437 (effect, −0.021; s.e.m., 0.002; 95% CI −0.017, 0.025; *P* = 7.599 × 10^−17^) and rs76761706 (effect, −0.035; s.e.m., 0.002; 95% CI −0.031, 0.039; *P* = 2.850 × 10^−40^) located in inversion regions on chromosome 8 and 17, respectively. Variants in these two inversion regions were also previously reported to be significantly associated with neuroticism in the study by Okbay et al.^12^.

For extraversion, after meta-analysing the MVP and GPC data, the number of significant loci increased to 14. The lead signals were located on chromosomes 1–6,11,12, 17 and 19. The most significant locus harbours genes in/near *WSCD2* (*P* < 3.449 × 10^−11^) located on chromosome 12.

**Fig. 1:**
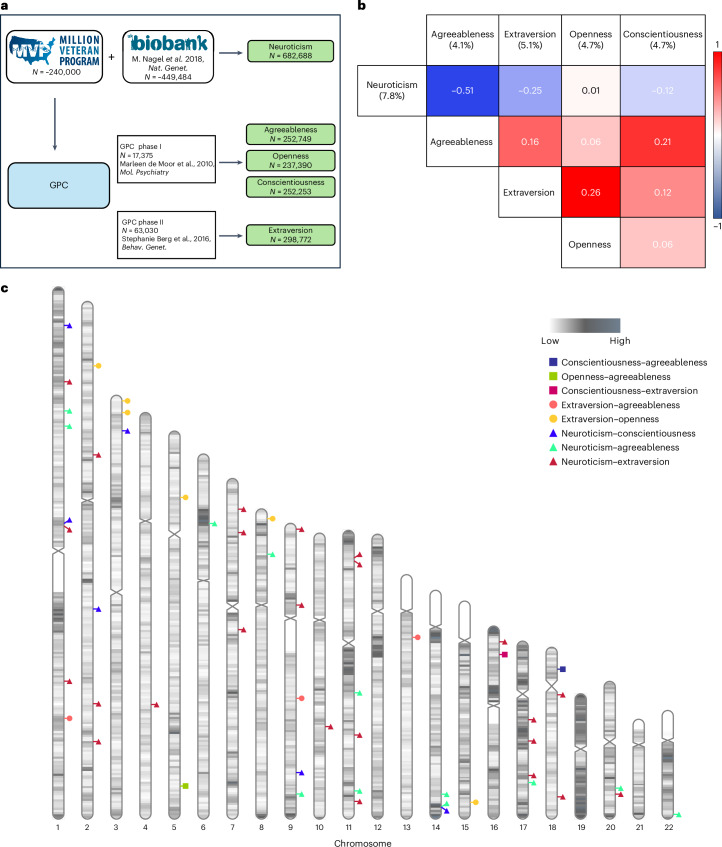
Personality GWAS meta-analysis and genetic correlations among the five personality traits. **a**, Data collection of the five personality traits. **b**, Genetic correlation matrix among the five personality traits (meta-data). The heritability value of the respective trait is written in parenthesis. **c**, A karyogram showing the regions with significant local genetic correlation (*r*_G_ > 0.3) between different personality traits.

**Fig. 2:**
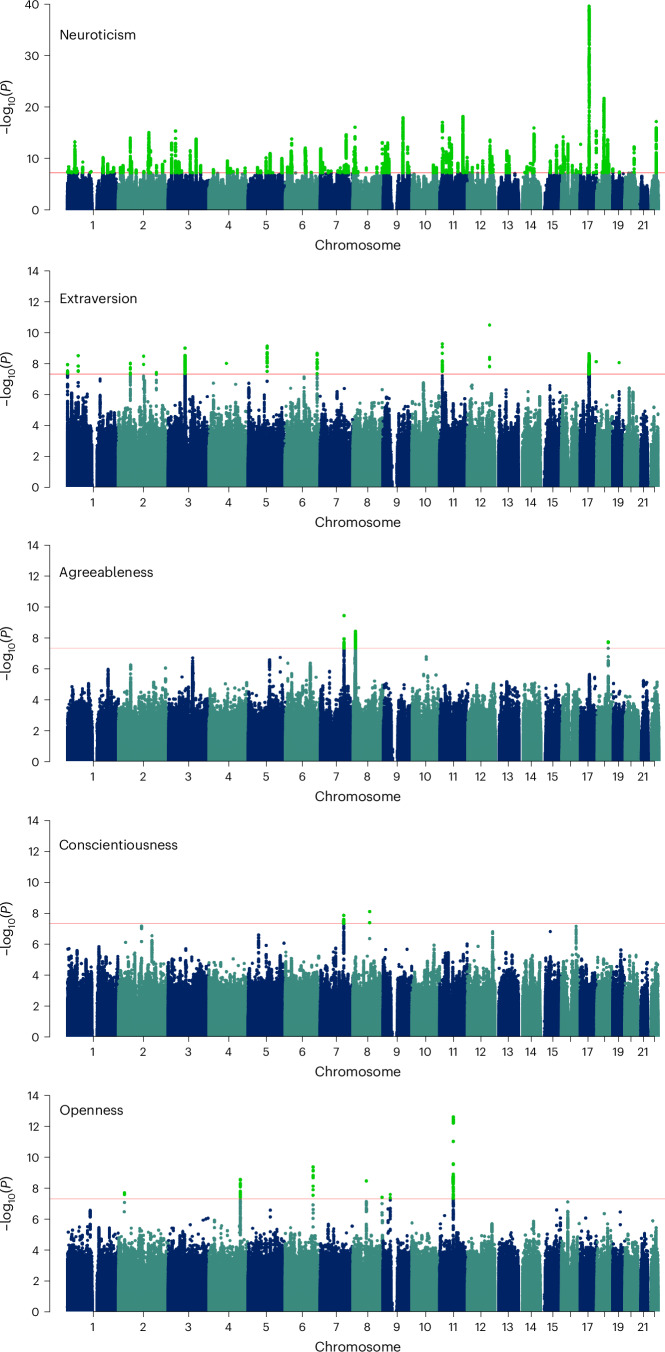
Meta-analysis data GWAS Manhattan plots of the five personality traits. The GWS variants in light-green colour. Reported *P* values are two-sided and not corrected for multiple testing. GWS threshold (*P* = 5 × 10^−8^) is used to define significant variants and depicted by red line.

Chromosome 11 contains significant variant associations from three traits, namely neuroticism, extraversion and agreeableness, with neuroticism and extraversion both having findings near the ‘basic helix-loop-helix ARNT like 1’ (*ARNTL1*, also known as *BMAL1*) gene, with opposing and significant direction of effect at common variants. Complete information of all identified significant loci for each of the five traits with full statistics is provided in Supplementary Tables 6–10. The cohorts used in meta-analysis are depicted in Fig. 1a. Manhattan plots for meta-analyses of each of the five traits are depicted in Fig. 2.

### Trans-ancestry analysis

We performed trans-ancestry meta-analysis of the five personality traits combining EUR and AFR GWAS for each of the five traits using inverse variance weighing in METAL^17^. For neuroticism, the trans-ancestry analysis identified a total of 216 GWS loci, of which 16 are novel, that is, they were not GWS in the EUR meta-analysis (Supplementary Tables 11–15). Of the 208 GWS loci for neuroticism in the EUR meta-analysis, 200 remained GWS in trans-ancestry analysis, while the remaining 8 showed a marginally higher *P* value and thus do not pass the threshold for being GWS in trans-ancestry. For agreeableness and conscientiousness, in addition to the loci that were shown to be GWS in their respective EUR meta-analysis, two more novel loci (rs140242735 located on chromosome 8 and rs10864876 located on chromosome 2 for agreeableness and conscientiousness, respectively) were identified as GWS in the trans-ancestry analysis. In case of openness, two loci out of the three that identified as GWS in EUR remained GWS in the trans-ancestry analysis. For extraversion, in total 13 were identified as GWS in the trans-ancestry analysis, of which 10 were also GWS in the EUR meta-analysis and 3 were newly identified.

### TWAS

**Fig. 3:**
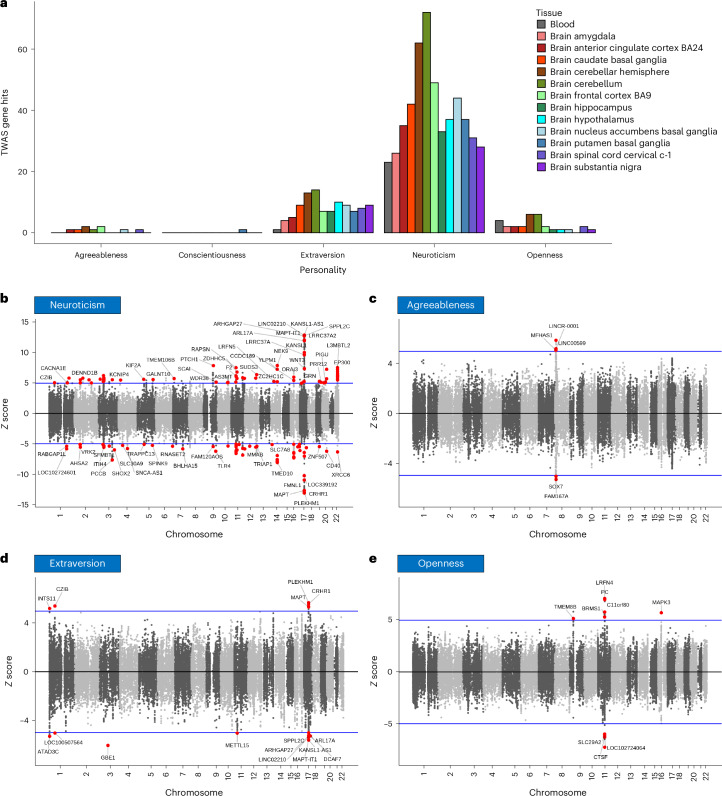
Transcriptome wide association study. **a**, A bar chart showing the number of significant TWAS genes per transcripts found of four personality traits with significant findings in respective subtissues. Scatter plots of neuroticism (**b**), agreeableness (**c**), extraversion (**d**) and openness (**e**) with TWAS *z*-scores of each gene transcript plotted on the *y* axis and its respective chromosomal location plotted on the *x* axis. The significant hits are shown in red circles with mapped gene names as labels. The blue horizontal line indicates the significance threshold of the *z*-score corresponding to the Bonferroni-corrected, two-sided *P* value. Conscientiousness data is reported in Supplementary Table 22.

We performed TWAS for each of the ‘big five’ personality traits in EUR (meta-analysis) using FUSION^18^ and the GWAS summary statistics. We performed a multi-tissue TWAS in 13 different brain subtissues and blood using their respective expression profiles from Genotype Tissue-Expression project (GTEx v8)^19^. From a total 10,386 genes tested, we identified a total 175, 24, 5, 1 and 11 genes showing significant gene–trait associations across the 13 subtissues in neuroticism, extraversion, agreeableness, conscientiousness and openness, respectively, after Bonferroni correction for 135,018 tests (10,386 genes across 13 tissues) (Fig. 3a). Figure 3a shows the distribution of associations found across the 13 tissues for each trait. The highest number of gene–trait associations were found in brain caudate basal ganglia, cerebellum, cerebral hemisphere and frontal cortex regions for neuroticism and extraversion, while fewer TWAS gene–trait associations were identified for the other three personality traits, presumably owing to the comparatively lower power of their respective GWAS datasets.

CRHR1, KANSLI1-AS1 and MAP-IT1 are among the top TWAS gene associations (*P* < 1.32 × 10^−23^) for neuroticism (Fig. 3b). The strong association of *CRHR1* (encoding corticotropic-releasing hormone receptor), which in some prior work has been shown to be associated with treatment response to depression^20^, may suggest some common underlying elements regulating both neuroticism and depression. Extraversion also shows strong gene–trait associations with CRHR1, KANSL1-AS1 and MAPT-IT1 but with an opposite direction of effect to neuroticism. This may indicate some common genetic components whose differential behaviour regulates neuroticism and extraversion. There are nine such genes showing opposite direction of effect in neuroticism and extraversion (Supplementary Table 3).

LOC10271024064 and LRFN4 showed the strongest associations with openness and LINCR-0001 and FAM167A showed the strong associations with agreeableness, while only one gene, *AP1G1*, showed association with conscientiousness in the 13 tissues considered. The complete list of all GWS TWAS gene hits for the five personality traits is provided in Supplementary Table 22.

### PWAS

We investigated the association of personality traits with protein expression using PWAS. Based on the availability of protein profiles and the observed TWAS signal, dorsolateral prefrontal cortex brain protein profiles were chosen for the PWAS analysis. The PWAS identified 47 proteins to be significantly associated with neuroticism. Next, we checked the colocalization signal for these PWAS lead genes. Out of 47 PWAS lead genes, 35 genes showed a colocalization signal (H4 probability >0.5).

**Fig. 4:**
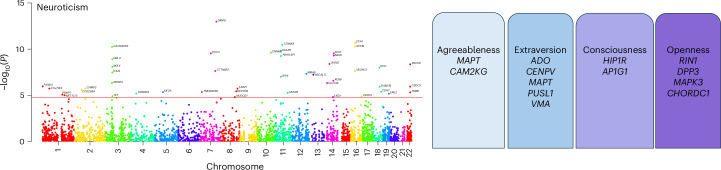
PWAS analysis. A Manhattan plot is displayed showing the significant protein associations observed for neuroticism. The red line in the plot depicts the Bonferroni-corrected, two-sided *P* value threshold at 5% FDR. The boxes on the right show the significant proteins found for the respective four personality traits.

Five, two, two and four proteins were discovered for extraversion, agreeableness, conscientiousness and openness, respectively (Fig. 4). A complete list of all PWAS lead genes is provided in Supplementary Table 23.

### LDSC

We first used LDSC to calculate SNP-based heritability of each of the five personality traits within the MVP EUR cohort. The intercepts of the LDSC indicated no evidence for population stratification, with observed values of 1.01, 1.02, 0.99, 1.02 and 1.00 for neuroticism, extraversion, agreeableness, conscientiousness and openness, respectively. The SNP heritability ranges from 4% to 7% (Supplementary Fig. 2), with extraversion showing the highest heritability point estimate of all traits (neuroticism *h*^2^ = 0.0655; s.e.m., 0.004; 95% CI 0.058, 0.073; agreeableness *h*^2^ = 0.042; s.e.m., 0.003; 95% CI 0.036, 0.048; extraversion *h*^2^ = 0.071; s.e.m., 0.003; 95% CI 0.065, 0.077; openness *h*^2^ = 0.048; s.e.m., 0.003; 95% CI 0.042, 0.054; and conscientiousness *h*^2^ = 0.047; s.e.m., 0.003; 95% CI 0.041, 0.053).

For the MVP AFR cohort, cov-LDSC was utilized to estimate personality heritabilities (Methods)^21^. Relative to the MVP EUR cohort, neuroticism and extraversion showed lower heritability (4.47% and 3.30%, respectively) in the AFR cohort, while for agreeableness, the heritability was similar (4.24%) (Supplementary Table 1). The values were not significant for conscientiousness and openness in AFR.

Before combining the MVP cohort-derived summary statistics with other data sources, we calculated the genetic correlation between the MVP personality summary statistics and other respective sources (Supplementary Table 2). A correlation coefficient value of 0.80 (s.e.m., 0.02) observed for the neuroticism summary statistics from the MVP cohort and Nagel et al. study^13^ suggests that there is limited heterogeneity between the two datasets and supports their use in a meta-analysis. As shown in Supplementary Table 2, the genetic correlations were high for all other four traits across data sources as well.

LDSC was used to estimate SNP-based heritability in the EUR participants for each personality trait in the meta-analysis. The SNP heritability values in the meta-analyses were similar to what was observed in the MVP-only cohort for the different traits in the EUR, with a decrease in heritability of extraversion from 7.1% to 5.1% (Fig. 1b).

Genetic correlation estimates were also obtained between the meta-analysis summary statistics for the five personality traits. We found a significant degree of varying genetic overlap among the five personality traits. The genetic correlations are presented in Fig. 1b. The highest correlation is observed between neuroticism and agreeableness with a *r*_G_ = −0.51 (s.e.m., 0.030; *P* = 3.813 × 10^−64^).

**Fig. 5:**
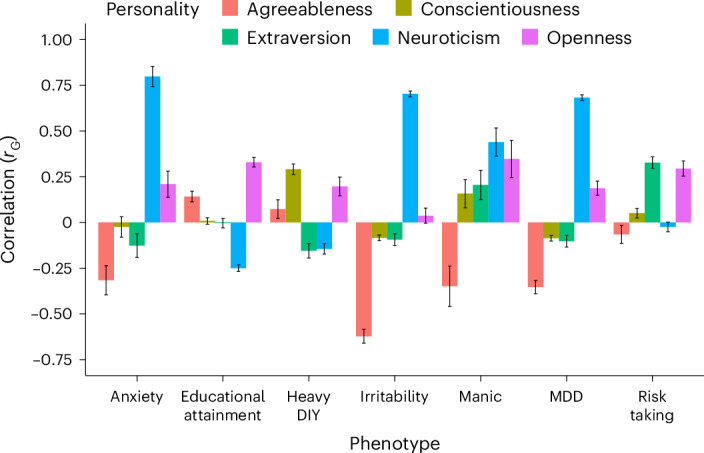
Bar chart with SNP-based genetic correlation of each of the five personality traits with a different psychological disorder/trait or different behaviours plotted. The *y* axis is the genetic correlation. Error bars (in black) indicate the 95% CIs of the estimated genetic correlation. Anxiety indicates substances taken for anxiety; medication is prescribed for at least 2 weeks. Heavy DIY activities describes the types of physical activity in last 4 weeks; for example, weeding, lawn mowing, carpentry and digging. Manic behaviour describes manic/hyper behaviour for 2 days. Detailed results for all traits, including the sample size of each of the traits, is presented in the Supplementary Tables 16–20.

Next, we estimated the genetic correlations of 1,437 traits listed in the Complex Traits Genetics Virtual Lab^22^ summary statistics record to find other traits related to the five personality traits (Supplementary Tables 16–20). A total of 325 traits showed significant genetic correlation following multiple testing correction to one or more personality traits. We found MDD and anxiety showed varying degrees of significant correlations to different personality traits as shown in Fig. 5. The highest genetic correlation is between neuroticism and anxiety (*r*_G_ = 0.80). Neuroticism and agreeableness both show high genetic correlations to these traits, but in opposite directions with MDD (neuroticism *r*_G_ = 0.68; s.em. 0.02; *P* < 5.00 × 10^−100^ and agreeableness *r*_G_ = −0.35; s.e.m. 0.04; *P* = 1.53 × 10^−22^), manic behaviour (neuroticism *r*_G_ = 0.44; s.e.m. 0.08; 95% CI 0.641, 0.719; *P* = 1.11 × 10^−8^ and agreeableness *r*_G_ = −0.35; s.e.m. 0.11; 95% CI −0.134, 0.566; *P* = 1.556 × 10^−3^), anxiety (neuroticism *r*_G_ = 0.80; s.e.m. 0.06; 95% CI 0.682, 0.918; *P* = 1.54×10^−46^ and agreeableness *r*_G_ = −0.32; s.e.m. 0.08; 95% CI −0.163, 0.477; *P* = 7.28 × 10^−5^) and irritability (neuroticism *r*_G_ = 0.70; s.e.m. 0.02; 95% CI 0.661; 0.739, *P* < 5.00 × 10^−100^ and agreeableness *r*_G_ = −0.62; s.e.m. 0.04; 95% CI −0.542, 0.698; *P* = 9.76 × 10^−61^).

### Local genetic correlations

Global genetic correlations use the average squared signal over the entire genome, which may sometimes mask opposing local correlations in different genomic regions. To counter that, we also calculated the local genetic correlations among the five personality trait pairs using Local Analysis of [co]Variant Association (LAVA)^23^. All personality pairs showed varying degree of correlation in different genomic regions except for the neuroticism–openness pair, which showed negligible global (*r*_G_ = −0.01) and no local genetic correlation between the two. The highest number of correlated genomic chunks were found for neuroticism–extraversion and neuroticism–openness pairs (Fig. 1c and Supplementary Table 21).

### Variant fine-mapping

To identify well-supported possible causal variants from the large list of SNPs showing associations with the personality traits, we performed genome-wide variant fine-mapping using PolyFun^24^. In total, 166 unique variants were fine-mapped across the five personality traits. The number of variants fine-mapped for neuroticism, extraversion, agreeableness, conscientiousness and openness were 155, 8, 4, 7 and 3, respectively. The complete list of variants fine-mapped for each of the personality traits is provided in the Supplementary Tables 24–28.

### Relationship between personality and psychiatric disorders

We performed additional analyses to help understand the significant differential genetic correlation observed between neuroticism and agreeableness with different psychiatric disorders such as MDD and anxiety.

### Conditional analysis

Because the genetic correlation between anxiety and neuroticism was so high, we performed multi-trait-based conditional and joint analysis of neuroticism summary statistics conditioned on anxiety and MDD summary statistics individually. The anxiety and MDD summary statistic used is based on data from UKB, MVP and PGC with individuals of EUR ancestry (see Methods for details). We performed a similar analysis with agreeableness, which had a negative correlation with both MDD and anxiety, as a negative control.

After conditioning on MDD, the SNP heritability of the conditioned neuroticism summary statistic reduced significantly from 7.8% to 3% (Table 2). Out of the original 208 GWS leads, only 42 remained significant after conditioning, indicating there is substantial genetic overlap between neuroticism and MDD, which gets removed after conditioning. In case of conditioning on anxiety, again there is a decrease in neuroticism heritability, but to a lesser extent (Table 2). On conditioning agreeableness on MDD and anxiety, no significant reduction in heritability was observed. However, loss of one genomic locus, rs7240986 (18:53195249:A:G), was observed after conditioning on either anxiety or MDD for agreeableness.

**Table 2:** Conditional analysis

Primary trait	Trait conditioned on	*h*^2^ (s.e.m.)	No. of GWS loci before conditioning	*h*^2^ (s.e.m.) after conditioning	*Z*-difference	*P* value	No. of GWS loci after conditioning
Neuroticism	Anxiety	0.078 (0.003)	208	0.05 (0.001)	8.85*	8.41e-19	96
Agreeableness	Anxiety	0.041 (0.003)	3	0.034 (0.003)	1.65	0.01	2
Neuroticism	MDD	0.078 (0.003)	208	0.03 (0.002)	13.31*	1.95e-40	42
Agreeableness	MDD	0.041 (0.003)	3	0.036 (0.003)	1.18	0.24	2

### Drug perturbation analysis

We performed a drug perturbation analysis to find drug candidates for neuroticism-enriched genes using gene2drug software^25^. Gene2drug utilizes the Connectivity Map transcriptomics data of ~13,000 cell lines exposed to different drugs, and based on these gene expression profiles and then pathway expression profiles (PEPs), it first matches the query gene to its pathway and then to its potential candidate drug. This analysis predicted 298 unique drugs to correspond to the 231 significantly associated neuroticism genes. The top-scoring drug was found to be desipramine, which is a tricylic antidepressant. Some of the other drugs predicted are flupenthixol (anti-psychotic), tetryzoline (α-adrenergic agonist), doxorubicin (anthracycline/chemotherapy) and digitoxigenin (cardenolide). Based on these results, we repeated the drug perturbation analysis with depression-enriched genes. While there were only 51 genes common between neuroticism and depression gene sets, there was a convergence on drugs in the perturbation analysis. Out of 286 and 298 drugs predicted for depression and neuroticism, respectively, 167 drugs were common to both. The complete list of drugs is presented in Supplementary Tables 29 and 30.

### MR

After establishing genetic overlap of neuroticism with MDD and anxiety, we carried out an MR analysis to explore the possibility of a causal relationship between genetic risk for neuroticism and MDD or anxiety. The results of the MR analysis using different methods are presented in Table 3. The results of MR indicate a bidirectional causal effect, with the exposure of MDD on neuroticism outcome showing an inverse variance weighting (IVW) effect value of 0.429 at a significant *P* value (2.072 × 10^−85^). The exposure of neuroticism on MDD shows a higher causal effect value of 0.834 with a significant *P* value (6.413 × 10^−103^). We performed sensitivity analysis of MR using MRlap, which corrects for different sources of bias, including sample overlap, because there are overlapping participants between the exposure and outcome datasets^26^. With MRlap, we observe similar results with positive significant corrected *β* values in MRlap performed between MDD and neuroticism in both directions (Supplementary Table 4).

**Table 3:** Outcome of MR experiments performed using MR

Trait	Two sample method	Exposure	Outcome	No. of instruments	*β*	*P*	Pleiotropy	Heterogeneity
Neuroticism	IVW	MDD	Neuroticism	71	0.429	2.072 × 10^−85^	3.48 × 10^−4^	248.350
	MR Egger				0.416	2.704 × 10^−5^		248.274
	Weighed mean				0.363	4.585 × 10−68		
	Simple mode				0.351	3.102 × 10^−9^		
	Weighed mode				0.336	3.427 × 10^−12^		
	IVW	Neuroticism	MDD	114	0.834	6.413 × 10^−103^	2.55 × 10^−5^	369.516
	MR Egger				0.791	7.795 × 10^−5^		369.340
	Weighed mean				0.734	3.418 × 10^−66^		
	Simple mode				0.748	1.729 × 10^−6^		
	Weighed mode				0.704	3.772 × 10^−6^		
	IVW	Anxiety	Neuroticism	75	0.179	1.248 × 10^−15^	0.007	389.419
	MR Egger				−0.002	9.585 × 10^−1^		307.410
	Weighed mean				0.101	1.182 × 10^−9^		
	Simple mode				0.081	9.700 × 10^−4^		
	Weighed mode				0.081	3.227 × 10^−3^		
	IVW	Neuroticism	Anxiety	126	0.700	5.767 × 10^−61^	−1.17 × 10^−3^	209.008
	MR Egger				0.766	3.174 × 10^−5^		208.767
	Weighed mean				0.706	8.209 × 10^−40^		
	Simple mode				0.821	2.764 × 10^−6^		
	Weighed mode				0.854	1.101 × 10^−7^		
Agreeableness	IVW	MDD	Agreeableness	66	−0.284	5.775 × 10^−13^	9.31 × 10^−4^	118.501
	MR Egger				−0.273	1.181 × 10^−1^		118.492
	Weighed mean				−0.281	5.703 × 10^−13^		
	Simple mode				−0.376	5.529 × 10^−3^		
	Weighed mode				−0.376	3.823 × 10^−3^		
	IVW	Agreeableness	MDD	32	−0.221	4.164 × 10^−6^	−1.17 × 10^−2^	133.267
	MR Egger				0.127	3.521 × 10^−1^		106.341
	Weighed mean				−0.172	6.621 × 10^−5^		
	Simple mode				−0.261	2.316 × 10^−2^		
	Weighed mode				−0.234	9.338 × 10^−4^		
	IVW	Anxiety	Agreeableness	68	−0.241	7.734 × 10^−16^	−5.40 × 10^−3^	102.166
	MR Egger				−0.112	1.135 × 10^−1^		96.094
	Weighed mean				−0.191	4.077 × 10^−7^		
	Simple mode				−0.155	7.727 × 10^−2^		
	Weighed mode				−0.172	4.346 × 10^−2^		
	IVW	Agreeableness	Anxiety	42	−0.224	1.157 × 10^−8^	−5.07 × 10^−3^	52.159
	MR Egger				−0.068	6.059 × 10^−1^		50.235
	Weighed mean				−0.198	1.436 × 10^−4^		
	Simple mode				−0.188	1.395 × 10^−1^		
	Weighed mode				−0.192	1.260 × 10^−1^		

We also investigated the casual relationship of neuroticism with anxiety. On performing MR with anxiety exposure on neuroticism, we found a *β* value of 0.179 (*P* = 1.248 × 10^−15^) and a corrected *β* value with MRlap of 0.531 (*P* = 7.781 × 10^−14^) showing evidence of causality. On reversing the direction, the causality effect was stronger as seen by higher *β* value of 0.70 (*P* = 5.767 × 10^−61^) with MR and corrected *β* value of 0.548 (*P* = 1.129 × 10^−40^) with MRlap. This suggests that there is stronger evidence of causal effect of neuroticism on anxiety as compared with the reverse based on the genetic susceptibility. GWAS of anxiety and anxiety disorders are still relatively underpowered compared with neuroticism, limiting the number of available genetic instruments available for testing as exposures.

We investigated the causal effect of agreeableness on MDD and anxiety and vice versa. In the case of MR of MDD exposure on agreeableness outcome, a *β* value of −0.284 (*P* = 5.775 × 10^−13^) was observed indicating negative causal effect of MDD on agreeableness (Table 3 and Supplementary Table 4). The causal effect is bidirectional with similar values observed in the opposite direction as well. The results are consistent with genetic correlation findings where negative correlation was observed between agreeableness and MDD. MR analysis of agreeableness and anxiety also indicated bidirectional causal effect. However, here both the traits have limited instruments available.

### Out-sample polygenic risk score prediction

We conducted polygenic prediction analysis to validate our findings using the Yale–Penn cohort^27^, which had NEO Personality Inventory (NEO PI-R) scores and genotype information available for 4,532 EUR individuals, and used those data to predict PRS for each of the big five personality traits (Methods). We found modest but significant *r*^2^ values in line with previous reports for all personality traits^14^: neuroticism of 2%, extraversion of 2%, openness of 2%, agreeableness of 3% and conscientiousness of 1%.

## Discussion

We conducted a GWAS meta-analysis study of each of the ‘big five’ personality traits in a sample size of up to 682,688 participants. We combined original GWAS results from the MVP (available for all five traits) with summary statistics from the UKB (neuroticism only) and GPC (all traits except neuroticism) cohorts to perform a well-powered meta-analysis for EUR GWAS in each trait. We identified 468 independent significant SNPs associations mapping to 208 independent genomic loci, of which one-third are novel. We identified 231 significant gene associations with neuroticism in the gene-based analysis. The current study was also successful in identifying 23 significant genomic locus associations for the four other personality traits studied, for which prior knowledge in the literature was very limited. In AFR, we found lower heritabilities for neuroticism and extraversion and no significant results for conscientiousness and openness. We identified two GWS variants for agreeableness in AFR. This is probably a reflection of low power and underlines the critical need to increase recruitment in underrepresented groups. Our work provides new data to inform the underlying genetic architecture of personality traits.

Neuroticism, the trait with the largest available sample size in this study, is characterized by emotional instability, increased anxiousness and low resilience to stressful events. As such, it has been the focus of previous efforts in GWAS. As seen previously, neuroticism overlaps substantially with psychopathology, where it is usually viewed as a precursor or risk factor for depressive and anxiety symptoms. Extraversion had the second largest sample size and had the highest SNP-based heritability in the MVP. In our data, scoring high on extraversion was genetically correlated with risk-taking behaviours and had the second strongest negative genetic correlation with neuroticism. Agreeableness assays show how someone relates with other people, that is, how trusting one is or how likely to find fault in others. This trait was the most negatively correlated with neuroticism and irritability as well as MDD, anxiety and manic symptoms. Conscientiousness items relate to discipline and thoroughness, with specific questions being ‘are you lazy’ and ‘does a thorough job’. This trait was most closely associated with ‘types of physical activity in last 4 weeks: ‘heavy do-it-yourself (DIY)’. Finally, openness 10-item Big Five Inventory (BFI-10) items assay imagination and artistic interest. Openness was positively associated with extraversion and risk taking in our data. Educational attainment was positively correlated with openness and negatively associated with neuroticism, while the other three personality traits showed essentially no such overlap (Fig. 5). Since these are self-reported items, they naturally reflect one’s own assessment of one’s personality traits, which might filter actual traits and behaviour through a lens of how one wishes to appear or be perceived.

Using these GWAS summary statistics, with excellent power for neuroticism and moderate power for the other traits, we investigated the heritability of the different personality traits and studied genetic correlations among them using LDSC. SNP-based heritability for all five personality traits in EUR were statistically significant. Out of all the personality pairs studied, the strongest relationship was a negative genetic correlation observed between neuroticism and agreeableness (*r*_G_ = −0.51, Fig. 1b). Examining the genetic correlations of the five personality traits with 1,437 external traits including depression (neuroticism *r*_G_ = 0.68 and agreeableness *r*_G_ = −0.35), manic behaviour (neuroticism *r*_G_ = 0.44 and agreeableness *r*_G_ = −0.35), anxiety (neuroticism *r*_G_ = 080 and agreeableness *r*_G_ = −0.33) and irritability (neuroticism *r*_G_ = 0.70 and agreeableness *r*_G_ = −0.62) further reflected a pattern of opposing relationships between these traits (Fig. 5 and Supplementary Tables 16–20). We also calculated local genetic correlations between personality pairs using LAVA, which helped in identifying the genomic regions playing roles in differential overlap in the genetic architecture of personality. This analysis identified several regions where the effect direction differed from the whole genome genetic correlation.

The MVP, our discovery dataset, is one of the world’s largest biobanks and is a valuable resource for genetic studies. Some previously published personality trait studies had significant contribution from UKB data. It is important to quantify the heterogeneity in these independent cohorts and the different definitions of personality phenotype within each. We investigated the genetic correlation between traits defined on the basis of different inventories (BFI-10, EPQ-RS and NEO-FFI) of personality ascertainment with different cohorts, namely MVP, UKB (part of Nagel et al. study) and GPC, respectively. For neuroticism, Nagel et al. and MVP studies showed a high *r*_G_ value of 0.80 making these two independent cohorts suitable for meta-analysis (Supplementary Table 1). Similarly, for extraversion, NEO-FFI and two-item inventories showed high *r*_G_ of 0.89 in the extraversion data of GPC and MVP studies. While for agreeableness, openness and conscientiousness, the *r*_G_s between MVP and GPC cohort were lower (0.63–0.72); this may be due to the small size of the GPC dataset for these traits and the correspondingly large standard errors around the point estimate. The point estimate is not necessarily biased in any particular direction, we only mean there is uncertainty. This limitation will be addressed by future GPC studies with larger sample sizes. No novel loci were identified in the meta-analysis with GPC for these traits.

TWAS revealed common genes with changes in gene expression but with opposite direction of effect for some personality traits. A study by Ward et al. in 2020 reported five of these genes (Supplementary Table 3) as eQTLs showing significant associations with mood instability^28^. This is further supported by the local genetic correlation studies (Supplementary Sheet 5) where we found genomic region 45883902-47516224 on chromosome 17, which harbours genes *KANSL1-AS1*, *MAPT* and *MAPT-IT1*, showing negative local genetic correlation between neuroticism and extraversion with a *ρ* value of −0.57 and *r*^2^ value of 0.32.

rs1876829, which maps to CRHR-Intronic Transcript 1, emerged as the lead SNP (*P* = 7.872 × 10^−39^) for neuroticism in the GWAS analysis. We also found multiple eQTL SNPs in this genomic region (rs8072451, rs17689471, rs173365 and rs11012) for the *CRHR1* gene to be significantly associated (*P* value ranging from 1 × 10^−5^ to 1 × 10^−37^). The TWAS analysis showed significant association of this gene with neuroticism in nervous system tissues including caudate basal ganglia, frontal cortex, hippocampus and spinal cord cervical region. *CRHR1* encodes the receptor of corticotropin-releasing hormone family, which are major regulators of the hypothalamic–pituitary–adrenal pathway^29^. Genetic variation in the corticotropin-releasing hormone system has been linked to several psychiatric illnesses^30^. Another study reported hypermethylation at corticotropin-releasing hormone-associated CpG site, cg19035496, in individuals with high general psychiatric risk score for disorders such as depression, anxiety, post-traumatic stress disorder and obsessive compulsive disorder^31^. Further, a study by Gelernter et al. found that *CRHR1* significantly associated with re-experiencing post-traaumatic stress disorder symptoms^32^ and also maximum habitual alcohol intake^33^. This gene is also involved in hippocampal neurogenesis^30^, while reduced hippocampal activation is associated with elevated neuroticism^34^. This makes *CRHR1* a good lead candidate to be followed in future studies to understand the molecular processes impacted by genetic variation underlying a range of psychiatric traits including neuroticism.

While gene expression associations give a wide array of information on the involvement of different genes regulating the different biological processes underlying the biology of traits, searching protein expression associations confers several advantages, as proteins are the final implementers in the functioning of all cells for many biological processes. Through PWAS studies, we found 47 proteins showing significant association with neuroticism in the dorsolateral prefrontal cortex. The PWAS analysis also identified leucine-rich repeat and fibronectin type III domain-containing 5 (LRFN5) protein association with neuroticism, and this protein is also involved in synapse formation. This protein has shown higher levels in patients with MDD and has been suggested as a potential MDD biomarker^35^.

Examples of genes for which we found converging evidence in neuroticism for transcript and protein-level associations with neuroticism include low-density lipoprotein receptor-related protein 4 (LRP4), syntaxin 4 (STX4) and metabolism of cobalamin associated B (MMAB) (Supplementary Table 31). LRP4 has diverse roles in neuromuscular junctions and in disorders of the nervous system, including Alzheimer’s disease and amyotrophic lateral sclerosis^36^, STX4 is implicated in synaptic growth and plasticity^37^, and MMAB, which catalyses the final step in the conversion of cobalamin (vitamin B12) into adenosylcobalamin (biologically active coenzyme B12), all of which have broad implications for brain function, including those in relation to methylmalonic acidaemia^38^. Low levels of plasma vitamin B12 have been found to be associated with higher depression cases in multiple studies^39^.

We investigated the relationship of these personality traits with other psychiatric traits, cognitive functions and disorders in a broad phenome-wide scan of genetic correlations with 1,437 traits. A total of 325 traits showed significant genetic correlations with at least one of the five personality traits following multiple testing correction. Two important traits that had some of the strongest associations were MDD and anxiety. Whereas the association of neuroticism with depression and anxiety has been previously considered^4,13^, our analysis revealed that another personality trait, agreeableness, is also strongly associated with both anxiety and depression but in the opposite direction to neuroticism, showing a potential protective relationship. MR indicated a strong bidirectional causal relationship between neuroticism with anxiety and depression, while showing a bidirectional protective relationship for agreeableness for both traits. Variance explained for neuroticism was attenuated upon conditioning for MDD but remained significant, indicating some independent genetic component for neuroticism despite the strong overlap. Similar, but with a less strong effect, was seen of anxiety on neuroticism, which may be partly due to lower power of available anxiety summary statistics. Larger studies of anxiety disorders are needed to better understand this relationship. Conversely, when we conditioned on agreeableness, for MDD and anxiety we observed a nominal but non-significant change in SNP-based heritability. We conducted MR to further discern these patterns and it showed bidirectional causal effects with neuroticism, confirming a high degree of inter-relatedness between the traits. Given the high degree of genetic overlap between trait neuroticism and the expectation of personality trait expression preceding age of onset for MDD, a high trait neuroticism may be considered an early risk factor for anxiety, depressive and related psychopathology. Indeed, studies have shown persistent elevated neuroticism through adolescence is a risk factor for later susceptibility to anxiety and MDD diagnosis^40^.

Personality phenotyping in The MVP sample were done using self-report for the short BFI-10 inventory. As such, data are relatively sparse compared with more robust instruments and do not have more in-depth features such as facets found in the NEO inventory. The nature of large biobank studies such as the MVP comes with a crucial advantage in recruitment and sample size, but comes with the sacrifice of deep phenotyping. Future studies that compare findings from more deeply phenotyped samples to more sparse phenotyping used by the MVP would be valuable to address this limitation. Additionally, while we greatly expand on the amount of data available for agreeableness, conscientiousness, openness and extraversion, they still lag behind what has been accomplished for neuroticism. This means genetic instruments defined for the other four traits may lack the precision available for neuroticism. Larger samples still need to be collected to better understand these other traits.

Personality traits are known to have complex interactions with other human behaviours. In this work we have conducted comprehensive genomic studies of personality traits. We performed a GWAS in the MVP sample, the largest and most diverse biobank in the world, in both EUR and AFR to better understand genetic factors underlying personality traits. We combined this information with previously published results in a large meta-analysis, identifying novel genetic associations with five personality traits studied. We identified interactions in a phenome-wide genetic correlation analysis, finding novel relationships between complex traits. We used in silico analysis techniques to identify genetic overlap and causal relationships with depression and anxiety disorders. We also characterized underlying biology using predicted changes in gene and protein expression, biological pathway enrichment and drug perturbation analysis. These results substantially enhance our knowledge of the genetic basis of personality traits and their relationship to psychopathology.

## Methods

### Inclusion and ethics statement

This research was not restricted or prohibited in the setting of any of the included researchers. All studies were approved by local institutional research boards and ethics review committees. MVP was approved by the Veterans Affairs central institutional research board. We do not believe our results will result in stigmatization, incrimination, discrimination or personal risk to participants.

### Cohort and phenotype

We used data release version 4 of the MVP^41^. The BFI-10 was included as part of a self-report Lifestyle survey provided to MVP participants, with two items for each of the personality traits (Supplementary Fig. 3). For the MVP EUR participants, the mean age was ~65.5 years for each of the five traits and 8% of the sample was female. For MVP AFR, the mean age was ~60.6 years for each trait while 14.0% of the sample was female.

### Genotyping and imputation

Genotyping and imputation of MVP subjects has been described previously^41,42^. A customized Affymetrix Axiom Array was used for genotyping. MVP genotype data for biallelic SNPs were imputed using Minimac4^43^ and a reference panel from the African Genome Resources panel by the Sanger Institute. Indels and complex variants were imputed independently using the 1000 Genomes phase 3 panel^44^ and merged in an approach similar to that employed by the UKB. Ancestry group assignment within the MVP has been previously described^45^. Briefly, designation of broad ancestries was based on genetic assignment with comparison to 1000 Genomes reference panels^44^. Principal components to be used as covariates were generated within each assigned broad ancestral group.

### GWAS and meta-analysis

We performed individual GWAS for each of the five personality traits in the MVP cohort^41^. The personality information along with genotype data were available for a total of 270,000 individuals with 240,000 EUR and 30,000 AFR. The GWAS was performed separately for each of the traits in the EUR and AFR datasets and the effect values were computed using linear regression.

MVP GWAS was conducted using linear regression in PLINK 2.0 using the first ten principal components, sex and age as covariates^46^. Variants were excluded if call missingness in the best-guess genotype exceeded 20%. Alleles with minor allele frequency (MAF) <0.1% were excluded. Additionally, only variants with an imputation accuracy of ≥0.6 were retained. After applying all filters, genotype data from 233,204, 235,742, 235,374, 234,880 and 220,015 participants were included for neuroticism, extraversion, agreeableness, conscientiousness and openness, respectively.

For meta-analysis, summary statistics generated in this study (referred to as MVP study) were combined using METAL^17^ with that from Nagel et al. and GPC phase I and II studies (Fig. 1a) based on the availability of data for respective traits. The *z*-scores of variants provided in the summary statistics were converted into *β* scores^47^. The inverse variance weighing scheme of METAL was applied to weight the effect sizes of SNPs from the different source studies. For neuroticism, summary statistics from MVP and Nagel et al. studies^13^ (excluding 23 and Me) were combined, increasing the total sample size to 682,688. For extraversion, summary statistics from MVP and GPC phase II study^10^ were combined, while summary statistics from MVP and GPC phase I study^8^ were combined for the respective meta-analysis of agreeableness, openness and conscientiousness. GPC data were already included in the neuroticism meta-analysis of Nagel et al.

The independent GWS loci for each of the personality traits were identified by clumping all SNPs using PLINK v1.9 software^48^. *P* value cut-off of 5 × 10^−8^, MAF >0.0001, distance cut-off of 1 MB and *r*^2^ < 0.1 were used to define the lead SNPs using the 1000 Genomes phase 3 European reference panel^44^. The genes are mapped for the identified lead SNPs using biomaRt package in R^49^. The same parameters were used to define novel independent loci for comparison from the Nagel et al.^13^ and Becker et al.^14^ summary statistics (excluding 23 and Me).

### Trans-ancestry analysis

Trans-ancestry analysis for each of the five personality phenotypes was performed by combining their respective summary statistics from AFR and EUR analyses using METAL^17^. As with the EUR-only meta-analysis, the inverse variance weighing scheme of METAL was applied to weight the effect sizes of SNPs from the two ancestries. We identified independent SNPs in the same manner as described above for the ancestry-stratified GWAS.

### LDSC and SNP heritability

LDSC was performed based on the linkage disequilibrium reference from the 1000 Genomes data for all EUR cohorts and SNP heritability for each of the five personality traits was calculated^50^. To investigate the relation among the different personality traits, the LDSC-based correlation was also calculated between each pair of traits^51^. LDSC was also used to calculate genetic correlation of the personality traits with multiple other phenotypes (1,437 traits) with the Complex Traits Genetics Virtual Lab webtool^22^. A *P* value cut-off of 6.9 × 10^−6^ (0.05/(1437 × 5)) was applied to filter the significant correlating pair of traits after multiple test correction.

For MVP AFR, linkage disequilibrium scores were computed from the approximately 123,000 AFR individuals’ genotype data in the MVP cohort using covariant LDSC software^21^. This linkage disequilibrium reference panel was then utilized to calculate SNP heritability in the MVP AFR cohort using LDSC.

### Local genetic correlations

We used LAVA^23^ to calculate local heritability for the five personality traits and local genetic correlations for each pair. The genome was divided into 2,495 genomic chunks/loci to attain minimum linkage disequilibrium between them and maintain an approximate equal size of around 1 MB. The local heritability of each of the five personality traits was calculated for each of the 2,495 loci. For a given personality trait pair, local genetic correlations were calculated only for pairs that had significant local heritability (Bonferroni-corrected *P* value at 5% false discovery rate (FDR)) for both traits of the pair. Bonferroni multiple testing correction was also applied to genetic correlated *P* value to consider significant correlated pairs.

### TWAS

FUSION software^18^ was used to perform TWAS. FUSION first estimates the SNP heritability of steady-state gene and uses the nominally significant (*P* < 0.05) genes for training the predictive models. The predictive model with significant out-of-sample *R*^2^ (>0.01) and nominal *P* < 0.05 in the five-fold cross-validation was then used for the predictions in the GWAS data. The process is performed for all five personality EUR GWAS data with 10,386 unique genes spanning over the 13 selected tissues. The expression weight panels for 13 a priori selected tissues were taken from GTEx v8^19^. We selected the different available brain tissues and whole blood as the tissues of interest, where Bonferroni corrections at FDR <0.05 were applied with the 10,386 genes test for the 13 tissues to find the genes with significant hits (P < 3.703 × 10^−7^).

### PWAS

We performed PWAS to test the association between genetically regulated protein expression and different personality traits individually using FUSION software^18^. The weights for genetic effect on protein expression for the PWAS were from the Wingo et al. study^52^. In the PWAS, we integrated the protein weights with the summary statistics from the GWAS of each of the personality traits, respectively. Next, to decrease the probability of linkage contributing to the significant association in the PWAS, we performed colocalization analysis using COLOC^53^. In COLOC, we determined if the genetic variants that regulate protein expression colocalize with the GWAS variants for the personality trait. Significant proteins in the PWAS that also have COLOC posterior probability of hypothesis 4 (PP4) >50% have a higher probability of being consistent with a causal role in the personality trait of interest.

### Fine-mapping

To identify likely causal variants, we performed variant fine-mapping using Polyfun software^24^. Since the fine-mapping was performed on the same EUR data, SNP-specific prior causal probabilities were taken directly from the pre-computed causal probabilities of 19 million imputed UKB SNPs with MAF >0.01 based on 15 UKB traits analysis. The fine-mapping was performed on the GWAS sumstats for each of the five personality traits. SuSiE^54^ was used to map the posterior causal probabilities of the SNPs. The SNPs with posterior inclusion probability (PIP) value >0.95 were considered as significant for neuroticism, while a more relaxed cut-off of PIP >0.80 was used for other four personality traits to avoid loss of causal variant information due to the relatively less power in their respective datasets.

### Conditional analysis

Conditional analysis was performed to investigate the possible mediating effects between depression or anxiety and neuroticism or agreeableness. Neuroticism meta-data GWAS summary statistics were used and conditioned on MDD and anxiety in individual runs. The MDD summary statistics were from Levey et al. study^55^ and include a meta-analysis from the MVP, UKB, PGC and FinnGen. The anxiety summary statistics were taken from Levey et al. study^42^. With depression/anxiety studies as covariate traits, the conditional analysis of neuroticism (target trait) was carried out using multi-trait-based conditional and joint analysis utility of genome-wide complex trait analysis^56^. Similarly, the same method was used to perform conditional analysis of agreeableness on MDD and anxiety.

### Drug perturbation analysis

FUMA was used to carry out the MAGMA-based gene-association tests to find significantly associated genes for a trait from its GWAS summary data^15^. Drugs were searched for both neuroticism and MDD individually using their respective significantly associated genes derived from neuroticism meta-analysis summary statistics and MDD GWAS summary statistics from the Levey et al. summary statistics. To predict drug candidates for a given trait, significant genes associated with neuroticism/depression were given as input to gene2drug R-package^25^. Pre-computed Pathway Expression Profiles of the Connectivity Map data were taken from Drug Set Enrichment Analysis (DSEA) website. For each query gene, a maximum of five predicted drugs were predicted. Further, the drugs showing an *E s*core >0.5 and a *P* value less than 1 × 10^−6^ were considered significant. The process was repeated for MDD.

### MR

MR was performed to study the causal relationship between four pairs of traits: neuroticism and MDD, neuroticism and anxiety, agreeableness and MDD, and agreeableness and anxiety. These traits had the highest genetic correlation. The summary statistics described previously for conditional analysis for all four traits were used for carrying out MR analysis as well. TwoSample MR package was used to perform the MR^57^. For each pair of traits, the TwoSample MR was run twice to see the effect of exposure of each of the two traits on the outcome of the other. After harmonizing the exposure and outcome instruments sets, clumping of SNPs (distance of 500 kb, *r*^2^ = 0.05) was performed before conducting the MR analysis. Because some of our samples included in the analysis of personality overlap with our outcomes and exposures of interest, and a TwoSample MR is not robust to sample overlap, we also performed a sensitivity analysis for each trait pair using the MRlap package^26^. MRlap is specifically designed to account for many assumptions of MR, including sample overlap. It first calculates observed MR-based effect values and then a corrected effect value by using the genetic covariance calculated by LDSC.

### Out-sample polygenic risk score prediction

The Yale–Penn cohort includes participants recruited from sites in the eastern United States^58^. A total of 11,705 participants completed the 240-item revised NEO PI-R, which assesses the domains of the five-factor model of personality: neuroticism, extraversion, openness to experience, agreeableness and conscientiousness^59^. Each domain has six facets. For example, the facets of neuroticism are anxiety, angry hostility, depression, self-consciousness, impulsiveness and vulnerability. Each item is rated on a five-point scale. Of the Yale–Penn participants with a NEO score, 4,582 were assigned to the broadly defined EUR group using the same methods as in the MVP sample and were unrelated. We used PRS-CS, Python software that uses Bayesian regression and continuous shrinkage priors, to calculate posterior effect sizes per SNP^60^. The 1000 Genomes linkage disequilibrium reference panel was used. The training datasets were summary statistics from the EUR meta-analysis for each of the five personality factors. The target dataset was a PLINK-formatted binary file set containing genotype information from the Yale–Penn participants^48^. Once score per SNP was generated by PRS-CS and PLINK was used to generate a score for each individual by summing SNP effect^48^. The lm (linear model) function in R was used to regress NEO PI-R scores on PRS, using age, sex and the first ten within-ancestry principal components as covariates^61^.

### Ethics oversight

Research involving MVP in general is approved by the Veterans Affairs Central institutional research board; the current project was also approved by institutional research boards in West Haven, CT.

### Reporting summary

Further information on research design is available in the Nature Portfolio Reporting Summary linked to this article.

## Supplementary information

Supplementary Information Supplementary Figs. 1–3, Tables 1–4 and VA Million Veteran Program core acknowledgement.

Reporting Summary

Supplementary Tables 1–31 Thirty-one supplementary tables.

## Data Availability

All MVP summary statistics are made available through dbGAP request at https://www.ncbi.nlm.nih.gov/projects/gap/cgi-bin/study.cgi?study_id=phs001672.v11.p1. Meta-analysis summary statistics are available through the Levey lab website at https://medicine.yale.edu/lab/leveylab/data/. Meta-analysis data will also be made available via the Complex Trait Genetics Virtual Lab at https://vl.genoma.io/.

## Code availability

No custom code was developed for analyses in this manuscript. All code used is cited and described in the methods. Software versions are accessible via PLINK v1.9 at https://www.cog-genomics.org/plink/1.9/, PLINK v2.0 at https://www.cog-genomics.org/plink/2.0/ and Polyfun: version 1.0.0 SuSiE package version: 0.11.92.
