# Supplementary material for: A genome-wide investigation into the underlying genetic architecture of personality traits and overlap with psychopathology
Source: Nat Hum Behav. 2024 Aug 12;8(11):2235–49. doi: 10.1038/s41562-024-01951-3 (PMC11576509; doi:10.1038/s41562-024-01951-3)
Supplement: Supplementary file 2 — Reporting Summary [file 41562_2024_1951_MOESM2_ESM.pdf]

nature portfolio

Corresponding author(s): Daniel F. Levey

Last updated by author(s): Jul 2, 2024

Reporting Summary

Nature Portfolio wishes to improve the reproducibility of the work that we publish. This form provides structure for consistency and transparency in reporting. For further information on Nature Portfolio policies, see our Editorial Policies and the Editorial Policy Checklist.

Statistics

For all statistical analyses, confirm that the following items are present in the figure legend, table legend, main text, or Methods section.

n/a	Confirmed	
☐	☒	The exact sample size (*n*) for each experimental group/condition, given as a discrete number and unit of measurement
☒	☐	A statement on whether measurements were taken from distinct samples or whether the same sample was measured repeatedly
☐	☒	The statistical test(s) used AND whether they are one- or two-sided<br>*Only common tests should be described solely by name; describe more complex techniques in the Methods section.*
☐	☒	A description of all covariates tested
☐	☒	A description of any assumptions or corrections, such as tests of normality and adjustment for multiple comparisons
☐	☒	A full description of the statistical parameters including central tendency (e.g. means) or other basic estimates (e.g. regression coefficient) AND variation (e.g. standard deviation) or associated estimates of uncertainty (e.g. confidence intervals)
☐	☒	For null hypothesis testing, the test statistic (e.g. *F*, *t*, *r*) with confidence intervals, effect sizes, degrees of freedom and *P* value noted<br>*Give P values as exact values whenever suitable.*
☒	☐	For Bayesian analysis, information on the choice of priors and Markov chain Monte Carlo settings
☒	☐	For hierarchical and complex designs, identification of the appropriate level for tests and full reporting of outcomes
☐	☒	Estimates of effect sizes (e.g. Cohen's *d*, Pearson's *r*), indicating how they were calculated

Our web collection on statistics for biologists contains articles on many of the points above.

Software and code

Policy information about availability of computer code

Data collection

No software was used for data collection.

Data analysis

PLINK was used for GWAS, METAL was used for meta-analysis, R was used for statistical tests, all R packages are mentioned explicitly in text where the package was used. FUSION software was used to perform TWAS using tissue databases described in text. GCTA was used for conditional analysis. All software packages used in this analysis are publicly available. R packages (MRlap) are cited and described in text.
FUSION does not report a version number.
Additional software versions:
PLINK v1.9 (https://www.cog-genomics.org/plink/1.9/),
PLINK v2.0 (https://www.cog-genomics.org/plink/2.0/),
Polyfun: Version 1.0.0
SuSiE package version: 0.11.92

For manuscripts utilizing custom algorithms or software that are central to the research but not yet described in published literature, software must be made available to editors and reviewers. We strongly encourage code deposition in a community repository (e.g. GitHub). See the Nature Portfolio guidelines for submitting code & software for further information.

Data

Policy information about availability of data

All manuscripts must include a data availability statement. This statement should provide the following information, where applicable:

- Accession codes, unique identifiers, or web links for publicly available datasets
- A description of any restrictions on data availability
- For clinical datasets or third party data, please ensure that the statement adheres to our policy

The GWAS summary statistics generated during and/or analyzed during the current study will be available via dbGAP; the dbGaP accession assigned to the Million Veteran Program is phs001672.v1.p. The website is: https://www.ncbi.nlm.nih.gov/projects/gap/cgi-bin/study.cgi?study_id=phs001672.v1.p1.
Meta-analyses will be made available additionally through other means to be cited in text at the time of publication.

Research involving human participants, their data, or biological material

Policy information about studies with human participants or human data. See also policy information about sex, gender (identity/presentation), and sexual orientation and race, ethnicity and racism.

Reporting on sex and gender	We have no available information on gender. Study design was population based -or- based on specific criteria (such as prior treatment in the US VA system) and efforts were made to recruit both males and females. We expect our results to apply to both sexes. The new part of the primary GWAS analysis involved the Million Veteran Program, which provided more than half of the total cases. This cohort is 91.9% male, making a stratification by sex challenging due to power differentials.
Reporting on race, ethnicity, or other socially relevant groupings	The MVP represents the one of the largest recruitments of non-European ancestries in the world. African ancestry makes up approximately 12% of the MVP sample reported here.
Population characteristics	The MVP is made of of veterans receiving care in the VA Healthcare System. Participants were 64.78 years old on average.
Recruitment	The Million Veteran Program (MVP) represents the majority of the sample. Active users of the Veterans Health Administration healthcare system (>8 million veterans) learn of MVP via an invitational mailing and/or through MVP staff while receiving clinical care with informed consent and HIPAA authorization as the only inclusion criteria. Enrollment involves providing a blood sample for genomic analyses, allowing ongoing access to medical records and other administrative health data by authorized MVP staff, and completing questionnaires.
Ethics oversight	Research involving MVP in general is approved by the VA Central IRB; the current project was also approved by IRBs in West Haven, CT.

Note that full information on the approval of the study protocol must also be provided in the manuscript.

Field-specific reporting

Please select the one below that is the best fit for your research. If you are not sure, read the appropriate sections before making your selection.

☒ Life sciences ☐ Behavioural & social sciences ☐ Ecological, evolutionary & environmental sciences

For a reference copy of the document with all sections, see nature.com/documents/nr-reporting-summary-flat.pdf

Life sciences study design

All studies must disclose on these points even when the disclosure is negative.

Sample size	Sample size reflected our best efforts to gather all possible participants with genetic data and available phenotypes as described.
Data exclusions	All subjects that passed basic quality control, were assigned to either European, African, Admixed American (name is from the reference panel which includes several groups collected from Latin American populations) and East Asian ancestry, and had available phenotype information were retained. All exclusion criteria were pre-established.
Replication	We performed genome-wide genetic correlations between all of the cohorts included in the meta-analysis. We used leave-one-out analysis to replicate previous findings from the field in the independent (and novel for the phenotype) MVP cohort
Randomization	Randomization was not applicable to this study. All available participants who responded to the Big Five personality survey items were included.
Blinding	Data were collected entirely independently of the analysts. There was no need for blinding or randomization.

Reporting for specific materials, systems and methods

We require information from authors about some types of materials, experimental systems and methods used in many studies. Here, indicate whether each material, system or method listed is relevant to your study. If you are not sure if a list item applies to your research, read the appropriate section before selecting a response.

Materials & experimental systems

n/a	Involved in the study
☒	☐ Antibodies
☒	☐ Eukaryotic cell lines
☒	☐ Palaeontology and archaeology
☒	☐ Animals and other organisms
☒	☐ Clinical data
☒	☐ Dual use research of concern
☒	☐ Plants

Methods

n/a	Involved in the study
☒	☐ ChIP-seq
☒	☐ Flow cytometry
☒	☐ MRI-based neuroimaging

Plants

Seed stocks	NA
Novel plant genotypes	NA
Authentication	NA
